# Cateslytin, a Chromogranin A Derived Peptide Is Active against *Staphylococcus aureus* and Resistant to Degradation by Its Proteases

**DOI:** 10.1371/journal.pone.0068993

**Published:** 2013-07-24

**Authors:** Rizwan Aslam, Céline Marban, Christian Corazzol, François Jehl, François Delalande, Alain Van Dorsselaer, Gilles Prévost, Youssef Haïkel, Corinne Taddei, Francis Schneider, Marie-Hélène Metz-Boutigue

**Affiliations:** 1 Inserm UMR-1121, Université de Strasbourg, Strasbourg, France; 2 EA-7290, Virulence bactérienne précoce, Fédération de Médecine Translationnelle de Strasbourg, Institut de Bactériologie, Université de Strasbourg – CHRU Strasbourg, Strasbourg, France; 3 CNRS UMR-7178, Institut Pluridisciplinaire Hubert Curien, Université de Strasbourg, Strasbourg, France; 4 Faculté de chirurgie dentaire, Université de Strasbourg, Strasbourg, France; 5 Service de Réanimation Médicale, Hôpital de Hautepierre, Université de Strasbourg, Strasbourg, France; Charité-University Medicine Berlin, Germany

## Abstract

Innate immunity involving antimicrobial peptides represents an integrated and highly effective system of molecular and cellular mechanisms that protects host against infections. One of the most frequent hospital-acquired pathogens, *Staphylococcus aureus*, capable of producing proteolytic enzymes, which can degrade the host defence agents and tissue components. Numerous antimicrobial peptides derived from chromogranins, are secreted by nervous, endocrine and immune cells during stress conditions. These kill microorganisms by their lytic effect at micromolar range, using a pore-forming mechanism against Gram-positive bacteria, filamentous fungi and yeasts. In this study, we tested antimicrobial activity of chromogranin A-derived peptides (catestatin and cateslytin) against *S. aureus* and analysed *S. aureus*-mediated proteolysis of these peptides using HPLC, sequencing and MALDI-TOF mass spectrometry. Interestingly, this study is the first to demonstrate that cateslytin, the active domain of catestatin, is active against *S. aureus* and is interestingly resistant to degradation by *S. aureus* proteases.

## Introduction

Chromogranins (Cgs) constitute the predominant family of proteins enclosed in secretory vesicles of chromaffin cells. [1] They are naturally processed to produce numerous peptides with various biological activities. [2]–[4] During the past decade, our group characterized several new antimicrobial peptides (AMPs) derived from chromogranin A (CgA) [5]–[8] and chromogranin B (CgB). [3], [9] These peptides are released by stimulated chromaffin cells of adrenal medulla and also by activated polymorphonuclear neutrophils (PMNs). [5], [7] Sequences of these peptides are highly conserved during evolution, suggesting that they are well-integrated in innate immune system. [10] Among these AMPs: chromofungin (CHR, CgA_47–66_) and catestatin (CAT, CgA_344–364_), derived from bovine CgA, activate PMNs and induce a calcium influx into immune cells. [11].

*Staphylococcus aureus* is the most frequently isolated pathogen in Gram-positive sepsis, often involved in blood clotting disorders and destruction of endocardial tissue. [12] *S. aureus* has developed several mechanisms to avoid immune response including resistance to AMPs, [13] impairment of phagocyte recruitment, [14] escape from neutrophil extracellular traps, [15] interference with complement, [16] neutrophil lysis, resistance to oxidative burst [17] and non-specific binding and degradation of immunoglobulins. [18] The AMPs evasion mechanisms deployed by *S. aureus* include proteolytic degradation by extracellular proteases of three major catalytic classes, namely metallo-, serine- and papain-like cysteine proteases. [19] The expression of proteolytic enzymes is controlled directly by global regulators of virulence factors such as *agr*, *sar* [20], [21] and indirectly by RsbU that controls Sigma(B) activity.[22] Moreover, *SarA* is also a regulator of methicillin resistance factor (fmtA).[23] It has been previously reported that *S. aureus* metallo-protease aureolysin can cleave and inactivate human cathelicidin LL-37, thereby contributing to bacterial escape from the innate immune system. [13].

As staphylococci easily colonize skin and epithelia, regardless of the expression of antimicrobial Cgs-derived peptides, [24] we aimed to investigate the antimicrobial effects of CAT and its shorter fragment cateslytin (CTL, CgA_344–358_) against *S. aureus*. Taking into account the difference in the activity of these peptides, using HPLC and proteomic analysis (sequencing and MALDI-TOF mass spectrometry), we examined the degradation of these CgA-derived peptides by staphylococcal proteases released into bacterial supernatants. Here, we report the relationship between peptide sequences and their sensitivity to bacterial proteases, as well as the possibility to use them as new antimicrobial agents in combination with antibiotics.

## Materials and Methods

### Preparation and analysis of synthetic antimicrobial peptides

Synthetic peptides were prepared on an Applied Biosystems 433A peptide synthesizer (Foster City, USA), using the stepwise solid-phase approach with 9-fluorenylmethoxycarbonyl (Fmoc) chemistry. Sequences of the synthetic peptides are as follows: bCAT (bCgA_344–364_: RSMRLSFRARGYGFRGPGLQL), hCAT (hCgA_352–372_: SSMKLSFRARGYGFRGPGPQL), bCTL (bCgA344–358: RSMRLSFRARGYGFR) and LL-37 (hCAP_134–170_ LLGDFFRKSKEKIGKEFKRIVQRIKDFLRNLVPRTES). Then, the synthetic peptides were purified by a Dionex HPLC system (Ultimate 3000; Sunnyvale, CA USA) on a Macherey Nagel Nucleosil RP 300–5C18 column (10×250 mm; particle size 5 µm and pore size 100 nm). Synthetic peptides were analysed by an automated Edman sequencing on an Applied Sequencing System Procise (Applied Biosystems, Foster City, USA). [9] Finally, MALDI-TOF mass measurements were carried out on an Ultraflex™ TOF/TOF (BrukerDaltonics, USA), as previously described. [25].

### Isolation and characterization of *Staphylococcus aureus* strains

Different *S. aureus* strains were used to demonstrate the peptide antimicrobial activity and subsequently, the peptide degradation: strains ATCC 25923, ATCC49775, S1 and S2, were provided by the Institute of Bacteriology, Strasbourg, France. S1 was isolated from the blood of an 83 y. o. patient and S2 was isolated from the *sputum* of a 12 days old neonate. After isolation and identification, *S. aureus* strains were assessed for their susceptibility to various antibiotics, using the agar disc diffusion method. [26] S1 was found Methicillin resistant (MRSA) and is also resistant to Amoxicillin, Oxacillin, Amikacin, Tobramycin, Fluoroquinolones, Erythromycin and Clindamycin. However, it was susceptible to Gentamicin, Synercid, Co-trimoxazole, Rifampicin, Fusidic acid, Vancomycin, Teicoplanin and Linezolid. In contrast, S2 was found to be Methicillin susceptible (MSSA) and is sensitive to all the antibiotics tested.

### Antibacterial activity against *S. aureus*

Different *S. aureus* strains described above were first pre-cultured aerobically at 37°C for 20 h in a Mueller-Hinton-Broth (MHB) medium, pH 7.3 (Difco Laboratories, Detroit, MI). Bacteria were suspended at absorbance of 0.001 at 620 nm in the MHB medium. Antibacterial activity was tested for 24 h incubation at 37°C with shaking by measuring the inhibition of bacterial growth. Ten µl final volumes (10–200 µg/mL) of synthetic peptides (LL-37, bCAT, hCAT and bCTL) were incubated in microtitration plates with 90 µl of a mid-logarithmic phase culture of bacteria, with a starting absorbance of 0.001 at 620 nm. In the initial inoculi, bacteria were quantified by the agar plate spreading method which was 5×10^5^ colony forming units (CFU)/mL. [27] Tetracycline (10 mg/L) and Cefotaxime (0.1 mg/L) were used as positive controls. Microbial growth was assessed by the increase of absorbance after 24 h incubation at 37°C. [28], [29] The A_620__nm_ value of control cultures growing in the absence of peptide and antibiotics was defined as 100% growth. A_620__nm_ zero with the antibiotics (Tetracycline and Cefotaxime) was taken as 100% inhibition. Absence of bacterial growth was verified by agar plate spreading. Each assay was performed in triplicates.

### Killing kinetics

Bacterial strains were first grown in MHB medium as described above. Bacteria killing kinetic activity was measured according to the previously described method, after making few modifications. [30] Initial inoculum was prepared at a concentration of 5×10^5^ CFU/mL with absorbance of 0.001 at 620 nm, which was calculated by agar plate spreading method. [27] Bacteria were incubated with different concentrations of peptides (MIC and 2× MIC), determined by MIC assay *via* microdilution method. [27] Viable bacterial count was then assessed at different time intervals up to 24 h. Briefly, aliquots were taken at different time intervals and were diluted in phosphate buffer saline (pH 7.4). After appropriate shaking 100 µL of the dilutions were plated on MH agar plates. Plates were incubated at 37°C and colony count was performed to determine CFU/mL after 24 h.

### Preparation of *S. aureus* supernatants

*S. aureus* strains were precultured and plated on the agar plates and cultivated for 24 h at 37°C. After incubation, one colony *per* isolate was transferred to 10 mL of the MHB medium and incubated at 37°C for about 30 h to late stationary growth phase. After incubation, cultures were centrifuged at 10,000×*g* for 15 min and supernatants were filtered using a 0.22 µM Millex®-GV (Millipore, Carrigtwohill, Ireland) to eliminate bacteria. The supernatants were stored at −20°C until further use. In order to check sterility, 1 mL of each supernatant was incubated at 37°C for 48 h. Absence of a colony was interpreted as lack of viable bacteria.

### Degradation analysis of the synthetic antimicrobial peptides by *S. aureus* supernatants

Synthetic peptides catestatin (bovine and human) and cateslytin (bovine) were incubated at 37°C for 24 h, with the different strain supernatants, at a concentration corresponding to the MIC values. Triplicate wells were treated for each concentration of peptide. In parallel, several controls were performed: with and without peptides in MHB medium.

In order to prevent degradation, the above experiment was repeated. For which, protease inhibitor cocktail (complete protease inhibitors except metalloproteases) (Roche Diagnostic GmbH, Mannheim, Germany) supplemented with 100 µM of 1–10 phenanthroline (metalloprotease inhibitor) (Sigma Aldrich GmbH, Steinheim, Germany) was added to bacterial supernatants before the addition of synthetic peptides. The whole suspension was incubated at 37°C for 24 h prior to being analyzed by RP-HPLC.

### RP-HPLC purification of CgA-derived peptides after incubation with *S. aureus* supernatants

The synthetic peptides treated with bacterial supernatants and different controls including culture medium, bacterial supernatants, and synthetic peptides were separated using a Dionex HPLC system (Ultimate 3000; Sunnyvale, CA USA) on a Nucleosil reverse-phase 300–5C18-column (4×250 mm; particle size, 5 µm; porosity, 300 Å) (Macherey Nagel Hoerdt, France). Absorbance was monitored at 214 nm, and the solvent system consisted of 0.1% (v/v) TFA in water (solvent A) and 0.09% (v/v) TFA in 70% (v/v) acetonitrile-water (solvent B). Elution was performed at a flow rate of 700 µL/min, with the gradient as indicated on chromatograms. Fractions were collected with one min time interval. They were subsequently concentrated by evaporation by using a speed-vac, however not allowing them to dry completely.

### Automatic Edman sequencing of CgA-derived peptides

The N-terminal sequence of purified peptides was determined by automatic Edman degradation analysis on a Procise microsequencer (Applied Biosystems, Courtaboeuf, France). Samples purified by HPLC were loaded on polybrene-treated glass-fiber filters. Phenylthiohydantoin-amino acids (Pth-Xaa) were identified by chromatography on a C_18_ column (PTH C-18, 2.1 mm×200 mm). [2].

### Proteomic analysis

Mass determination was carried out on a Brucker BIFLEX™ Matrix-Assisted Laser Desorption Ionization – Time-Of-Flight mass spectrometer (MALDI-TOF) (equipped with the high resolution optics (SCOUT™) with X–Y multi-sample probe, a gridless reflector and with the HIMAS™ linear detector). With a maximum accelerating potential of 30 kV, the system can be operated either in linear or the reflective mode. Ionization was carried out with a 337-nm beam from a nitrogen laser with a repetition rate of 3 Hz. The output signal from the detector was digitized at a sampling rate of 250 MHz in linear mode and 500 MHz in reflector mode using a 1 GHz digital oscilloscope (Lecroy model). Brucker supplied software hosted in Sun sparcworkstation was used for data processing and the instrumental control. [31] These studies were realized using the matrix α-cyano-4-hydroxycinnamic acid, obtained from Sigma, and prepared as a saturated solution in acetone. A total of 1–2 µl of the sample matrix aliquot solution was deposited on the probe and dried by ambient air. A thin layer matrix crystal was obtained after rapid spreading and evaporation. [32] A micromolar analyte solution was applied to the matrix and allowed to dry under moderate vacuum. The whole preparation was washed by 1 μl of trifluoroacetic acid (0.5%) aqueous solution. This cleaning procedure helps remove remaining alkaline cations and often leads to an increase in sensitivity.

### Statistical analysis

The MIC values (µg/mL) are reported as means of three independent experiments. To determine significance, between different peptides (rows) and bacterial strains (columns), one way ANOVA was used. Overall significance and correlation was evaluated by 4×4 factorial ANOVA for independent samples. Significance was accepted at *p*≤0.05.

## Results

### Antibacterial activity of CgA-derived peptides against *S. aureus*

**Figure 1:**
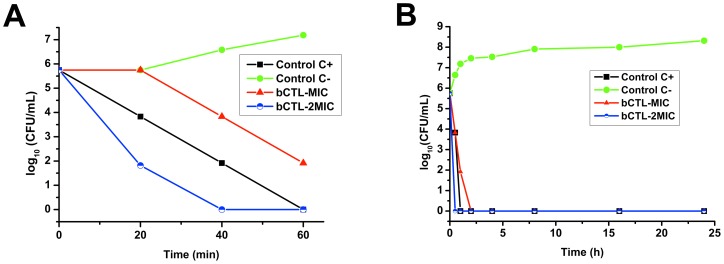
The bacterial killing kinetics of the bCTL against the *S. aureus* ATCC 25923. Different concentrations (MIC 40 µg/mL and 2× MIC 80 µg/mL) of bCTL were used. C+, represents antibiotic control (Cefotaxime 0.1 µg/mL + Tetracycline 10 µg/mL) and C-, represents phosphate buffer saline control. (A): *S. aureus* killing kinetics at time zero to 60 min. (B): *S. aureus* killing kinetics over 24 h time period.

The aim of present study was to examine antibacterial activity of bovine and human CAT (bCgA_344–364_ and hCgA_352–372_) and bovine CTL (bCgA_344–358_), against *S. aureus*. In comparison with the other peptides tested, CTL was found to be most active against all *S. aureus* strains tested (ATCC49775, ATCC25923, S1, and S2). Activity of these CgA derived peptides was compared to the well-known C-terminal peptide (LL-37) of hCAP-18 (Table 1). Activity of bCTL was comparable to that of LL-37 (*p*>0.05) against S1 and S2, but significantly different (p<0.05) for ATCC49775 and ATCC25923. However, MIC (presenting 100% growth inhibition) values for bCAT and hCAT were highly significantly different (*p*<0.01) that were about 2–4 fold higher than short fragment CTL (Table 1). MIC value for CTL varies between different *MRSA* and *MSSA* strains (*p*<0.05) and can effectively kill bacteria at 45 µg/mL. While both human and bovine CAT have more than 100 µg/mL MIC values. In order to detail CTL antimicrobial activity, we further determined the killing rate of CTL. Time-killing kinetic assays were performed against *S. aureus* (ATCC 25923). As shown in Figure 1, CTL acts very rapidly against *S. aureus*. AT concentration 2×MIC CTL present 4 logs bacterial killing at within 20 min of treatment and 100% within 40 min (Figure 1A). Moreover, at MIC, CTL reaches 100% bacterial killing within 60 min of treatment. This killing assay was continued till 24 h (Figure 1B) and bacteria were unable to grow further during 24 h. As CTL is the most active, we hypothesized this fragment is more resistant to proteases produced by *S. aureus*. In order to demonstrate the stability of CTL compared to human and bovine CAT, we incubated the three peptides with the supernatants of four different *S. aureus* strains.

**Table 1:** Antibacterial assays of catestatin (bovine, bCAT and human, hCAT) and cateslytin (bovine, bCTL) against different *S. aureus* strains, compared to the cathelicidin antimicrobial peptide-18 (LL-37).

Antimicrobial peptides	MIC (µg/mL)			
	*S. aureus* ATCC49775	*S. aureus* ATCC25923	*S. aureus* S1 (MRSA)	*S. aureus* S2 (MSSA)
bCAT	100 ^aA^	100 ^aA^	100 ^aA^	115 ^bA^
hCAT	125 ^aB^	135 ^aB^	130 ^aB^	150 ^bB^
bCTL	37 ^aC^	40 ^aC^	37 ^aC^	45 ^bC^
LL-37	30 ^aD^	30 ^aD^	30 ^aC^	35 ^bC^

Results are presented as MIC (µg/mL) of each peptide against four *S. aureus* strains. Values represent the means of the triplicate (n = 3) wells. Means with same letters are not significantly different (*p*<0.05). Small letters (a, b, c) represents significance between different *S. aureus* strains, while capital letters (A, B, C) represent significantly difference between peptides.

### Analysis of the proteolytic cleavage of catestatin (h/bCAT) and cateslytin (CTL) in the presence of *S. aureus* strain supernatants

**Figure 2:**
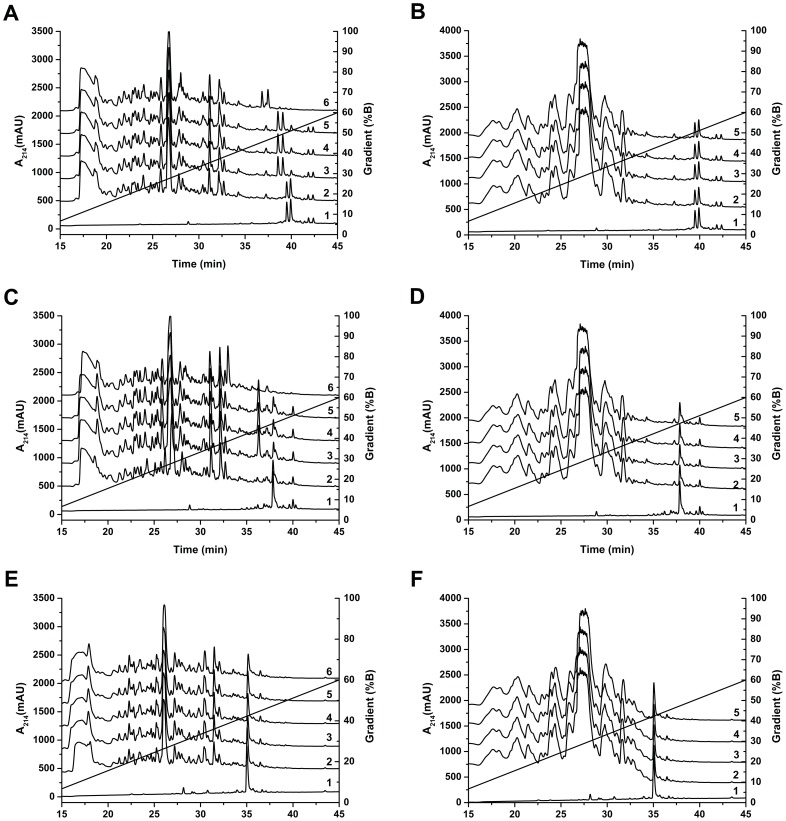
HPLC chromatograms of bCAT, hCAT and CTL alone or with different bacterial strain supernatants, with or without protease inhibitors. (A): Alignment of the HPLC chromatograms corresponding to: (1) bCAT, (2) bCAT+MHB, (3) bCAT+S49775, (4) bCAT+S25923 (5) bCAT+S1 (6) bCAT+S2. (B): Alignment of the HPLC chromatograms corresponding to: (1) bCAT, (2) bCAT+Pi+S49775, (3) bCAT+Pi+S25923 (4) bCAT+Pi+S1 (5) bCAT+Pi+S2. (C): Alignment of the HPLC chromatograms corresponding to: (1) hCAT, (2) hCAT+MHB, (3) hCAT+S49775, (4) hCAT+S25923 (5) hCAT+S1 (6) hCAT+S2. (D): Alignment of the HPLC chromatograms corresponding to: (1) hCAT, (2) hCAT+Pi+S49775, (3) hCAT+Pi+S25923 (4) hCAT+Pi+S1 (5) hCAT+Pi+S2. (E): Alignment of the HPLC chromatograms corresponding to: (1) bCTL, (2) bCTL+MHB, (3) bCTL+S49775, (4) bCTL+S25923 (5) bCTL+S1 (6) bCTL+S2. (F): Alignment of the HPLC chromatograms corresponding to: (1) bCTL, (2) bCTL+Pi+S49775, (3) bCTL+Pi+S25923 (4) bCTL+Pi+S1 (5) bCTL+Pi+S2.

*S. aureus agr* and *sar* regulate expression of proteases, which often are modulated by *SarA* regulator during biofilm formation. [33] These proteases are mostly expressed in the late growth phase and secreted in the extracellular environment to facilitate the bacterial spread. [34] As described above, *S. aureus* supernatants were prepared by the extended incubation period and are rich in proteases. Four *S. aureus* strains were tested for these experiments: two of them are referenced strains (ATCC 49775 and ATCC 25923), while two strains (S1 and S2) were isolated from the patients of Strasbourg hospital. S1 is a *MRSA* (methicillin resistant *S. aureus*) and S2 is a *MSSA* (methicillin sensitive *S. aureus*). The synthetic peptides were incubated with *S. aureus* supernatants and were separated by RP-HPLC (Figure 2 A, C, E). The HPLC profiles of CAT and CTL are compared with HPLC profiles of MHB medium and *S. aureus* supernatants. Peaks resulting from the bacterial degradation were analyzed by automatic Edman sequencing and MALDI-TOF. In contrast to bovine and human CAT (Figure 2A, 2C), CTL is not degraded (Figure 2E).

**Figure 3:**
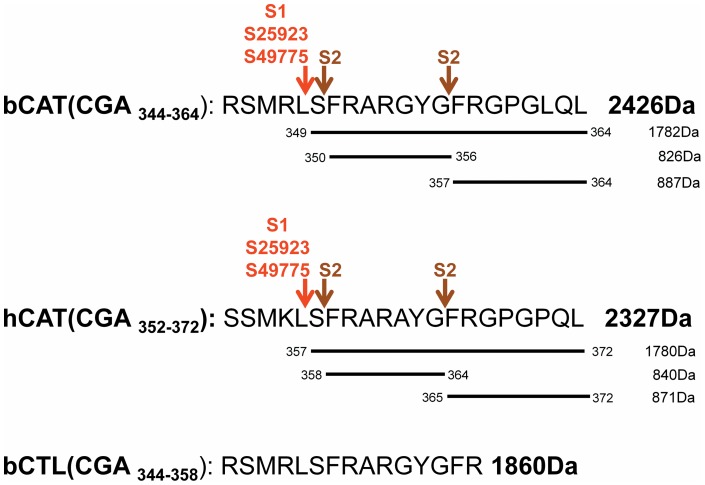
The proteolytic cleavage sites induced after treatment of bCAT, hCAT and CTL with different *S. aureus* strains (ATCC25923, ATCC49775, S1 and S2). The experimental molecular masses obtained after MALDI-TOF analysis, are indicated.

HPLC of bCAT (Figure 2A) indicates 2 major peaks, eluted at 38.7 and 40 min, corresponding to full-length peptide according to sequencing and MALDI-TOF mass spectrometry (2426 Da) (Figure 3). Presence of these 2 isoforms may be related to beta-turn conformation induced by proline residue in position 360 and its isomeric state (cis/trans). [35] In MHB medium, bCAT was not processed. Whereas, in presence of culture supernatants (S49775, S25923 and S1), bCAT was processed to generate fragments eluted at 38.3 and 38.6 min (Figure 2A). Sequencing and MALDI-TOF analysis indicate that these 2 fragments correspond to the isoforms of bCgA_349–364_ (1782 Da) (Figure 3). In presence of culture supernatant of S2, bCAT was largely cleaved to generate fragments eluted at 28.0, 36.6 and 37.5 min (Figure 2A). Sequencing and MALDI-TOF analysis indicate that these fragments correspond to bCgA_350–356_ (826 Da) and the 2 isoforms of bCgA_357–364_ (887 Da) (Figure 3).

Moreover, the HPLC profile of hCAT (Figure 2C) showed one major peak at 38 min that corresponds to the full-length peptide according to sequencing and MALDI-TOF mass spectrometry (2327 Da) (Figure 3). In presence of MHB medium, hCAT was not cleaved. However, after incubation with S49775, S25923 and S1, hCAT was partially processed to generate a fragment eluted at 36 min. Sequencing and MALDI-TOF analysis indicated that this fragment corresponds to hCgA_357–372_ (1780 Da) (Figure 3). In presence of supernatant S2, hCAT was completely cleaved to generate fragments eluted at 28.5 and 33 min (Figure 2C). Sequencing and MALDI-TOF analysis indicated that these fragments correspond to hCgA_358–364_ (840 Da) and hCgA_365–372_ (871 Da) (Figure 3).

Finally, the shortest peptide bCTL (bCTL, CgA_344–358_), is eluted as a single peak (Figure 2E) and is not degraded by any of the staphylococcal strains tested.

Interestingly, incubation of synthetic peptides with bacterial supernatants supplemented with protease inhibitors, prevents the peptide degradation (Figure 2B, D, F). HPLC profiles of bCAT (Figure 2B: chromatogram 2–5) indicate that it is not cleaved by any of the four strain supernatants tested in presence of protease inhibitors. Similar results were obtained for hCAT (Figure 2D: chromatogram 2–5). In addition, CTL remains unaffected by *S. aureus* supernatants with or without protease inhibitors (Figure 2E, F).

The proteolytic cleavage sites of bCAT and hCAT obtained after incubation with S49775, S25923 and S1 were identical (Figure 3). They corresponded to the peptide bonds L_348_−S_349_ and L_356_–S_357_ for bCAT and hCAT, respectively. With S2, they correspond to the peptide bonds S_349_−F_350_+G_356_−F_357_ and S_357_−F_358_+G_364_−F_365_ for bCAT and hCAT, respectively. It is important to point out that the short peptide bCTL (bCgA_344–358_) derived from CAT was not cleaved by *S. aureus* proteases (Figure 2E, F) demonstrating that CTL resists to the proteolytic cocktail produced by *S. aureus*.

## Discussion

Due to continuous resistance development by *S. aureus,* the interest for natural AMPs is increasing because some of them display potent antibacterial activity. However, secondary reactions such as hemolytic activity, [36] limit their use as systemic anti-infective drugs. Until recently, no such reaction was reported for the antimicrobial Cgs-derived peptides in the literature. Several CgA-derived peptides were previously characterized to display antimicrobial properties, [9] however, data concerning killing of *S. aureus* is not reported. CAT was previously reported, to be active against different bacterial strains at micromolar range. [24] CTL is well characterized for antimicrobial activity against *Micrococcus luteus*, few yeasts and fungal strains. Moreover, it is not hemolytic for human erythrocytes. [5]. In the present study, for the first time, we brought to light the antibacterial activity of CTL against different strains of *S. aureus* with a MIC value of 37–45 µg/mL (Table 1).

*In vivo*, a cross talk is established *via* dynamics of the interactions between host and bacteria. Many bacterial strains express a variety of proteases, ranging from non-specific and powerful enzymes that degrade many proteins involved in innate immunity, to proteases that are extremely specific in their mode of action. [37] Here, we questioned whether *S. aureus* proteases can cleave the three CgA-derived peptides studied. This proteolytic degradation could explain the 3–4 fold difference observed in antibacterial activity. To address this issue, we hypothesized that the human and bovine CAT peptides are degraded by the staphylococcal proteases and that CTL resists to the degradation and maintains its activity. Therefore, we tested the effect of four staphylococcal supernatants and demonstrated that bovine and human CAT are similarly degraded to generate inactive fragments, while CTL is not degraded. For bovine and human CAT, the cleavage sites induced by the supernatants are different. For S2 these correspond to the cleavage of S-F/G-F. Whereas, for ATCC49775, ATCC25923, and S1, it corresponds to cleavage of L-S (Figure 3). Upon cleavage by *S. aureus* proteases, the CAT peptides are no longer active against *S. aureus.* Previous data showed that bovine CgA_344–351_ and CgA_348–358_ display an antimicrobial activity against *Micrococcus luteus* at 20 µM, suggesting that L348/356 is important for its antibacterial activity. [5] Furthermore, in the protease inhibition assay, we demonstrated that degradation of human and bovine CAT can be prevented by the use of protease inhibitors. CTL maintains its position either with or without protease inhibitors indicating that proteases have no effect on the CTL.

The biosynthesis of CTL results by the action of prohormone convertases PC1/2 present in the granular matrix of chromaffin cells. [2], [38] The prohormone thiol protease (PTP) was also reported to be essential for CAT synthesis (bCgA_344–364_) by cleaving D-R and L-R. [38] In addition, in chromaffin secretory vesicles, the cysteine protease cathepsin L (CTSL) [39] generates CTL by the additional cleavage R-G of CAT. CAT fragment is known to activate the neutrophils by inducing extra-cellular calcium influx in human neutrophils *via* calmodulin-regulated calcium independent phospholipase A2. [11] Here, we demonstrate that CTL is resistant to the degradation by staphylococcal proteases, which strengthens the involvement of this CgA-derived domain in innate immunity. [10], [40].

**Figure 4:**
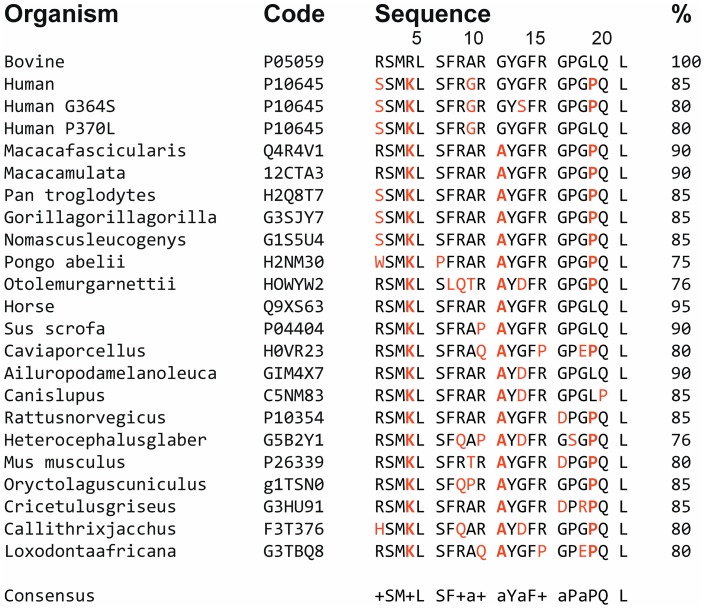
Sequence alignment of bovine catestatin (CgA_344–364_) with corresponding fragments from several species. For each position predominant identical residues are indicated in bold letters. Homology sequence is indicated (%).The data base used is UniProtKB. (+, basic residue; a, for A/G/T/P).

The primary structures of CAT and CTL are highly conserved during evolution (Figure 4). Arginine ratio of these peptide sequences is important, since it modulates the interaction with negatively charges of the microorganism's membranes. In addition, it was previously shown that arginine residues have a high tendency to interact with the lipids as suggested for other peptides, such as HIV-1 transcriptional activator Tat protein. [41], [42] For hCAT, bCAT, and bCTL the arginine ratios are 15%, 23%, and 33% respectively. High arginine ratio of bCTL supports a strong interaction with the negatively charged lipid bilayer as compared to the both CAT. Structure activity relationship of CTL with bacterial membrane is also demonstrated by recent experiments concerning the CTL derived peptide with a hydrophobic N-terminal end FLE-CTL (FLE-RSMRLSFRARGYGFR) (5R). With the help of HPLC and antimicrobial assays, we have shown that this synthetic peptide is inactive against *S. aureus* at 400 µg/mL and interacts strongly with bacterial membrane. In contrast, the shorter peptide that is lacking the C-terminal end (YGFR) of CTL: FLE-RSMRLSFRARG, displays antibacterial activities at 200 µg/mL (data not shown). Thus, CTL sequence possesses the cationic amphipathic features to exhibit potent antimicrobial activities.

Concerning its secondary structure, an aggregated antiparallel beta sheet structure was previously reported for CTL [43] by CD (Circular Dichroism) and ATR (Attenuated total reflectance) experiments, whereas hCAT and bCAT form short helical structures (residue 7 to residue 11) in the presence of high concentrations of DPC, as confirmed by CD and NMR (Nuclear magnetic resonance) experiments. [44] CTL adopts a major β-sheet character only on negatively charged membranes, whereas it is essentially unstructured in water. These β-sheet formations give a much more stability to peptide than the helical formation reported for CAT. [44] In addition, the (HR-MAS) ^1^H NMR analysis of CTL indicates that the arginine and hydrophobic residues are in close proximity, thus creating a deep penetration of charged residues into the membrane. [42] Therefore, the electrostatic interaction between positively charged arginine residues and negatively charged lipids appears to be responsible for binding of CTL to the lipid bilayer and the aromatic residues stabilize the lipid-peptide interaction.

In our group, we recently evaluated the synergistic effect of three CgA-derived peptides (CAT, CTL and amidated CTL) with antibiotics and demonstrated that this co-treatment induce a reduction of the antibiotics concentration used and can potentiate their activities. Antimicrobial assays were carried out by combining the AMPs at concentration below the MIC. The comparison was made with antibiotic or peptide separately at the same doses. For all these experiments, we evaluated the Fractional Inhibitory Concentration (FIC) of CgA-derived peptides combined with Minocyclin or Voriconazole. FIC corresponds to a synergistic effect in the range of ≤0.5, an additive effect when it is between >0.5 and <2, and an antagonistic effect when it is >2. [45] For the combination of amidated CTL and Minocyclin, we obtained a FIC of 0.37 against *S. aureus,* and for CTL and Voriconazole, we obtained a FIC of 0.25, and 0.5 against *Candida albicans* and *Candida tropicalis* respectively. [10] Regarding these data, one could imagine a mechanism in which, the peptides could favor destabilization of bacterial membrane, thus allowing the antibiotics to rapidly penetrate inside bacterial cells to reaching their site of action. These *in vitro* results are likely to occur *in vivo*, especially during systemic inflammation. Indeed, sepsis triggers numerous changes in proteases and inhibitors activity. [46] These regulations have been strongly related with sepsis severity. [47] Under such influences, CAT and CTL could even be difficult to release, owing to structural modifications of CgA, [48] due to sepsis-related oxidative stress. These modifications include oxidation of methionine, aromatic residues, glycanes, phosphorylation and also aggregation of the complete protein that might prevent the processing of CgA to produce active antimicrobial peptides. The structure-function relationship of AMPs is also very important to unravel the regular pattern of antibacterial activity. It has been previously demonstrated, that selective end tagging or short hydrophobic stretches can be, added to increase AMPs activity. [49], [50] Recently, we studied the properties of a modified CTL by inserting cysteine at C-terminal. This modified CTL was used to develop antimicrobial polymer coatings *via* alternative deposition of polyelectrolytes conjugated to CTL-C. [51] Activity of CTL is not altered, even with this modification or when inserted into PEM (Polyelectrolyte multilayer) coatings. Moreover, it is non-toxic for human gingival fibroblast. [51].

To conclude, for the first time we present the involvement of staphylococcal proteases on the cleavage of antimicrobial CgA-derived peptides. As the CTL domain is resistant to staphylococcal proteases, it constitutes a promising natural antibacterial peptide for further studies.
